# The Impact of Recovered Lignin on Solid-State PEO-Based Electrolyte Produced via Electrospinning: Manufacturing and Characterisation

**DOI:** 10.3390/polym17070982

**Published:** 2025-04-04

**Authors:** Laura Coviello, Giorgia Montalbano, Alessandro Piovano, Nagore Izaguirre, Chiara Vitale-Brovarone, Claudio Gerbaldi, Sonia Fiorilli

**Affiliations:** 1 Department of Applied Science and Technology (DISAT), Politecnico di Torino, Corso Duca Degli Abruzzi 24, 10129 Torino, Italy; laura.coviello@polito.it (L.C.); giorgia.montalbano@polito.it (G.M.); chiara.vitalebrovarone@polito.it (C.V.-B.); claudio.gerbaldi@polito.it (C.G.); 2 National Reference Centre for Electrochemical Energy Storage (GISEL)–INSTM, Via Giusti 9, 50121 Firenze, Italy; 3 Chemical and Environmental Engineering Department, Engineering Faculty of Gipuzkoa, University of the Basque Country UPV/EHU, Plaza Europa 1, 20018 Donostia, Spain; nagore.izaguirre@ehu.eus

**Keywords:** PEO, solid polymer electrolyte, lignin, electrospinning, lithium battery

## Abstract

Lithium batteries have gained significant attention due to their high energy density, specific capacity, operating voltage, slow self-discharge rate, good cycle stability, and rapid charging capabilities. However, the use of liquid electrolytes presents several safety hazards. Solid-state polymer electrolytes (SPEs) offer a promising alternative to mitigate these issues. This study focuses on the preparation of an ionically conductive electrospun membrane and its potential application as an SPE. To support a circular approach and reduce the environmental impact, the target polymeric formulation combines poly(ethylene oxide) (PEO) and lignin, sourced from paper industry waste. The formulation is optimised to ensure the dissolution of lithium salts and enhance the membrane integrity. The addition of lignin is crucial to contrast the dendrites’ growth and prevent the consequent battery breakdown. The electrospinning process is adjusted to obtain stable, homogeneous nanofibrous membranes, which are characterised using electron scanning microscopy (SEM), Fourier-transform infrared spectroscopy (FTIR), X-ray diffraction (XRD), differential scanning calorimetry (DSC), and thermal gravimetric analysis (TGA). The membranes’ potential as an SPE is assessed by measuring their ionic conductivity (>10^−5^ S cm^−1^ above 50 °C) and anodic stability (≈4.6 V vs. Li/Li^+^), and by testing their compatibility with lithium metal by reversible cycling in a symmetric Li|Li cell at 55 °C.

## 1. Introduction

Rechargeable energy storage devices (e.g., lead–acid, nickel–metal hydride, and lithium-ion batteries) are in high demand in a wide range of electric applications, from household appliances to electric vehicles, and from computers and smartphones to stationary energy storage systems coupled with renewable sources [1]. Lithium-ion batteries (LIBs) are distinguished by their high energy density due to high specific capacity and operating voltage, limited self-discharge rate, good cycle stability, and fast charging [2,3]. Based on the reported characteristics, they are employed in a multitude of sectors, including the powering of electric vehicles, power grid systems, and portable devices [4]. Standard commercial LIB cells consist of the combination of an anode, a cathode, and a separator soaked by the electrolyte, where the operational principle is based on the electrochemical reactions between separate electrodes, which are supported by the electrolyte as a conductive medium for the lithium ions. During the charging process, the cathode transfers lithium ions to the anode via the electrolyte through a separator. Subsequently, the anode completes the circuit by transferring electrons to the cathode, whereas the process is reversed during the discharge (spontaneous) phase. It then becomes clear how both the electrolyte and the separator exert a significant influence on the final performance of the battery. Although LIBs are commercially available and broadly diffused nowadays, commonly used liquid electrolytes are linked with several potential safety risks, including poor heat dissipation, leakage, and the uncontrolled growth of lithium dendrites. The occurrence of such issues can lead to the development of short circuits, fires, and explosions, with the subsequent evolution of toxic gases, raising some concerns about safety and environmental impact [1,5,6,7,8].

To overcome these limitations, solid-state electrolytes (SSEs) have been proven to represent a valid alternative thanks to their excellent flexibility, good chemical stability, tunable shape and size, longer lifetime, and light weight while hindering the growth of lithium dendrites, thereby enhancing the safety of the battery [1]. An additional advantage of using SSEs is the potential simplification of the internal structure, rendering the assembly process more straightforward and, thus, reducing the associated costs [6].

Solid-state electrolytes can be classified into two categories: solid-state inorganic ceramics and solid-state polymer electrolytes (SPEs). Inorganic ceramics demonstrate high electrical conductivity but suffer from inadequate mechanical properties (being rigid and brittle) and poor interfacial contact with the electrode. On the other hand, SPEs, while generally exhibiting lower ionic conductivity at room temperature, show exceptional plasticity, flexibility, and easy preparation. These characteristics make SPEs an attractive technology for potential industrial scaled-up production and suitable for commercial applications, particularly at elevated temperatures (T > 60 °C) [1,9,10].

SPEs are typically produced in the form of thin polymer films by using standard casting techniques. Nevertheless, this methodology is not without its inherent challenges, including volume shrinkage, uncontrolled thickness, and the persistence of solvent residues [11]. In recent years, electrospinning (ESP) has proven potential as a promising alternative manufacturing technique, enabling the fabrication of highly porous and flexible nanofibrous membranes characterised by a high surface-to-volume ratio and by a controlled entanglement of polymer chains thanks to the rapid evaporation of the solvent, features that play a key role in the transfer of ions between electrodes, thus improving the conductivity [11,12,13]. The ESP process involves the deposition of dry fibres from extruded polymer solutions by applying an electric field, where both solution and process parameters can be optimised to exert partial control over the deposition and morphological characteristics of the membranes [6,14]. In several application sectors, including regenerative medicine and water filtration, electrospinning (ESP) is utilised as a simple, versatile, cost-effective, and industrially scalable technique to produce nanofibrous membranes, which are also available as commercial products. ESP has also been proposed as a manufacturing technique in the energy sector, mainly to produce anodes and cathodes, and, more recently, nanofibrous polymer separators [5,6,15,16].

To date, several polymers have been evaluated for their suitability to produce electrolyte membranes, including poly(ethylene oxide) (PEO), poly(acrylonitrile) (PAN), poly(methylacrylate) (PMA), poly(methylmethacrylate) (PMMA), poly(vinylidene fluorideco-hexafluoropropylene) (PVDF-HFP), polyphenyleneoxide (PPO), brominated poly(phenylene oxide) (BPPO), and their combination [17]. PEO is a semi-crystalline polymer characterised by a high dielectric constant that has been extensively studied for the realisation of SPEs especially due to its ability to dissociate alkali metal salts and to form a stable interface with most of the electrodes [17]. As a matter of fact, PEO can effectively accommodate lithium ions by complexing them with the oxygen atoms of the ether groups, and it exhibits a good ionic conductivity (especially at high temperatures, close to its melting point) via a combination of segmental movement of the polymer chains (favoured by an appropriate interatomic distance between the oxygen atoms) and the hopping mechanism of Li^+^ ions (coordinated but not significantly retained by the ether groups) [6,17,18,19].

Up to now, a range of lithium salts has been investigated for use in PEO-based electrolytes of which the most frequently reported are hexafluorophosphate (PF_6_^−^), tetrafluoroborate (BF_4_^−^), perchlorate (ClO_4_^−^), triflate (CF_3_SO_3_^−^), bis(trifluoromethanesulfonyl)imide (TFSI^−^), and bis(pentafluoroethanesulphonyl)imide (BETI^−^). Among these, lithium bis(trifluoromethane)sulfonimide (LiTFSI) was comprehensively explored in different studies due to its intrinsic characteristics, including (i) the plasticising effect, thus reducing the crystallinity of PEO, (ii) the highly delocalised charge distribution of the anion, which leaves the Li^+^ cation free to move in the polymer matrix, and (iii) the excellent thermal, chemical, and electrochemical stability [20]. These properties make LiTFSI an optimal choice for the fabrication of ionically conductive and reliable SPEs, which can be employed not only in lithium batteries but also in a plethora of other devices [20].

Despite the combination of PEO and LiTFSI having been already explored in the literature for the design of SPEs, the resulting systems showed poor mechanical strength and poor resistance to Li dendrite growth, which is a frequent cause of short circuits and battery failure [21].

One potential polymer for reinforcing PEO membranes is lignin, a complex macromolecule that, along with cellulose and hemicellulose, forms the structural basis of plant cell walls, so that it is widely abundant in nature and frequently obtained as a side product in the paper industry. Its high carbon content, excellent thermal stability, and favourable stiffness make it a particularly appealing option to improve the chemical stability and mechanical strength of polymer matrices [5].

Among the various types of lignin, which are primarily categorised based on the extraction technique used, kraft lignin accounts for approximately 85% of the lignin available industrially [5,22,23,24]. Compared to the native lignin in wood, kraft lignin is a polydisperse material with a lower molecular mass, which consequently exhibits higher solubility making its use more versatile [25]. Moreover, compared to other industrial sources, it is characterised by a higher degree of purity due to the low presence of inorganic impurities and carbohydrate residues and overall greater mechanical strength.

The presence of numerous polar groups in lignin endows it with several highly promising chemical and physical characteristics. A review of the literature reveals that in recent years, lignin has begun to be studied for processing via ESP, specifically with the aim of manufacturing battery separators. However, lignin is also capable of dissociating lithium salts and influencing the ion transport process, suggesting its potential application in the production of SPEs [3].

To our knowledge and according to the existing literature, the ESP-based development of functional SPEs reinforced with natural polymers has yet to be explored. Notably, while numerous studies have explored the realisation of PEO-based SPEs using electrospinning, they often fall short by not demonstrating the final applicability of the resulting membranes in electrochemical cells. This is primarily due to the low mechanical strength of the polymeric system, which can be significantly improved by introducing lignin. In addition to creating polymeric membranes with enhanced mechanical performance, the development of an innovative formulation incorporating recovered materials aligns well with the principles of a circular economy, where waste is minimised, and materials are repurposed to gain renewed value.

Accordingly, this work aims at obtaining an electrospun nanofibrous membrane with the ability to conduct lithium ions, potentially serving as an SPE, exploiting the combination of PEO and lignin. The polymer concentration, ratio, and solvents were accurately optimised to ensure the processability of the new formulation with ESP technology by assessing the material rheology and different process parameters. Subsequently, the resulting electrospun membranes were comprehensively characterised via scanning electron microscopy (SEM), Fourier-transform infrared spectroscopy (FTIR), X-ray diffraction (XRD), differential scanning calorimetry (DSC), and thermal gravimetric analysis (TGA), particularly exploring the effects of lignin introduction on the final properties of the material. As the final step to prove the potential of the developed system as an SPE, the polymer membrane was tested for its ionic conductivity and electrochemical behaviour is symmetrical Li│Li battery test cells.

## 2. Materials and Methods

### 2.1. Materials

Poly(ethylene oxide) (PEO), with an average molecular weight (M_w_) of 200,000 g/mol, Acetonitrile (≥99.9% GC), and Lithium bis(trifluoromethane)sulfonimide (LiTFSI) were all purchased from Sigma-Aldrich (Merk KGaA, Saint Louis, MA, USA). Lignin was derived from paper and wood wastes through a Kraft process [26], by treating the biomass with NaOH and Na_2_S, precipitating the lignin by adding H_2_SO_4_ up to pH = 2, and finally neutralising and washing the precipitate with distilled water. The polymers were dried under a dynamic vacuum at 50 °C for one night before being stored within an Ar-filled glove box. LiTFSI, instead, was outgassed at 110 °C.

### 2.2. Preparation of Polymeric Solutions

**Figure 1:**
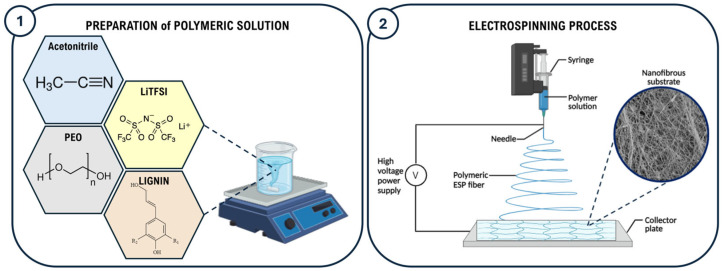
Schematic illustration of the preparation of the polymeric formulation (**1**) and manufacturing of the electrospun SPE membrane (**2**).

To prepare the electrospinning solution named PEO-Lignin-LiTFSI, PEO, lignin, and LiTFSI powders were weighed inside a glove box and fully dissolved in acetonitrile by applying magnetic stirring for 3 hours at room temperature (RT). The formulation consists of 10% *w*/*v* PEO, with lignin added to achieve a PEO-to-lignin ratio of 9:1. LiTFSI was added to achieve a LiTFSI-to-synthetic-polymer ratio of 1:10, as schematically represented in Figure 1. A lignin-free solution, designated PEO-LiTFSI, was also prepared and used as a control in this study.

### 2.3. Rheological Analysis

The rheological properties of the polymeric solutions were measured using a DHR-2 controlled-stress rotational rheometer (TA Instruments, Waters, New Castle, IN, USA) equipped with a 25 mm diameter parallel plate geometry and a Peltier system for temperature control. The viscosity was measured using a flow ramp test, at a constant temperature of 23 °C (ESP average working temperature) and varying the shear rate between 10 s^−1^ and 1000 s^−1^. Tests were performed in triplicate and data are presented as mean values.

### 2.4. Preparation of Solid Polymer Electrolytes (SPEs)

The electrospun membranes were produced using a commercial Fluidnatek LE-50 electrospinning machine (Bioinicia, Valencia, Spain). As shown in Figure 1, the polymer solutions were loaded into a 6 ml syringe using a 21 G needle (0.8 mm internal diameter) kept at a constant distance of 18 cm (working distance) from the flat collector, covered with aluminium foil to facilitate membrane removal. To achieve the stabilisation of the polymeric jet, different combinations of parameters were tested especially by varying the flow rate and voltage values in the ranges of 0.3–2 mL/h and 15–19 kV, respectively. Furthermore, a negative voltage of −2 kV was applied to the collector to improve the fibre deposition. The temperature and relative humidity in the ESP chamber were constantly monitored during the process, registering values in the ranges of 23–29 °C and 33–53%, respectively. The final membranes were obtained upon 4 hours of processing to obtain a final thickness in the range of 30–100 μm, with a corresponding area density of ca. 1.5–2 mg cm^−2^. The electrospun matrices were left drying overnight at room temperature, then outgassed at 40 °C for two days under a dynamic vacuum and finally stored in an Ar-filled glove box before use.

### 2.5. Characterisation of Solid Polymer Electrolytes (SPEs)

A detailed morphological analysis of the resulting fibrous membrane was carried out via scanning electron microscopy (SEM, Phenom XL, Phenom-World B.V., Eindhoven, The Netherlands). Samples of the electrospun membranes were placed on 12 mm diameter stubs covered by carbon tape and then sputter coated with a 7 nm platinum layer using a Cressington Sputter Coater 180 machine (Cressington, Watford, UK). Following image acquisitions, the fibre distribution and size were determined by using the ImageJ software (v. 1.54).

Infrared spectroscopy (FTIR) was conducted at room temperature using a Bruker Optics FTIR Tensor 27 Spectrometer (Bruker, Billerica, MA, USA) in Attenuated Total Reflection (ATR) mode (with a ZnSe crystal), covering a range of 4000 cm^−1^ to 600 cm^−1^. The spectra were acquired over 64 scans with a resolution of 2 cm^−1^. X-ray diffraction measurements (XRD) (Xpert, Philips, Malvern Panalytical Ltd., Malvern, UK) were performed employing CuKα radiation emitted at 40 kV and 40 mA. The corresponding data were collected from 2θ = 10° to 80°, with a step size of 0.013 2θ degrees and a scan step time of 45 s. The analysis enabled the determination of the proportion of the crystalline phase in the membranes by exploiting the following equation:

(1) $$\mathrm{Crystalline}\;\mathrm{phase}\left(\%\right)=\left(\frac{\mathrm{Total}\;\mathrm{area}\;\mathrm{of}\;\mathrm{crystalline}\;\mathrm{peaks}}{\mathrm{Total}\;\mathrm{area}\;\mathrm{of}\;\mathrm{all}\;\mathrm{peaks}}\right)\times 100$$

The thermal properties of the polymeric ESP membranes were characterised using differential scanning calorimetry (DSC) and thermal gravimetric analysis (TGA). The DSC was performed in a temperature range between 25 °C and 350 °C using a heating rate of 10 °C/min under a nitrogen atmosphere. Meanwhile, TGA was conducted from 20 °C to 600 °C with a heating rate of 8 °C/min in a nitrogen atmosphere.

### 2.6. Electrochemical Characterisation

All the electrochemical characterisations were performed in laboratory-scale electrochemical battery test cells (model ECC-Ref, EL-cell, Hamburg, Germany) using a VMP3 potentiostat/galvanostat (Biologic, Grenoble, France). In particular, the ionic conductivity was measured by interposing the membranes between two stainless steel blocking electrodes and recording electrochemical impedance spectroscopy (EIS) analysis (frequency from 100 kHz to 1 Hz) in the temperature range between −10 and 60 °C, whereas the electrochemical stability window (ESW) was assessed at 55 °C via linear sweep voltammetry (LSV) in a cell with metal Li and carbon-coated Al as electrodes at a scan rate of 0.1 mV s^−1^. The reversible Li plating and stripping tests were carried out at 55 °C in a symmetrical Li|Li configuration with an alternating current of 50 mA cm^−2^ for 2 hours in each verse.

## 3. Results and Discussion

### 3.1. Optimisation of Polymeric Solution and Electrospinning of the SPEs

The processing of PEO-based formulations using electrospinning for the design of SPEs for lithium batteries has been described in the literature, with a particular focus on the process setup and composition, including the PEO molecular weight, concentration, and salt ratio [6,10,27]. However, the few available studies all lack a demonstration of the actual feasibility of using the resulting electrospun (ESP) membrane as effective solid electrolytes in cells, likely due to the well-known poor stability of the PEO systems [17,18,28,29].

In this work, the authors aimed at contributing to fill this gap by developing a new PEO-based formulation and subsequently a solid polymer electrolyte (SPE) capable of ensuring efficient ion exchange and enhanced stability within the cell. Additionally, lignin, a natural polymer derived from industrial waste, was selected as the stabilising material, promoting sustainable solutions in line with a circular economy. Indeed, lignin is an excellent option for a circular economy because it is a feedstock from biorefineries, available worldwide and largely abundant, not competing with food and feeds (as starch and sugar), and exploiting the same infrastructure already established for the paper and pulp industry [30]; up to now, the main limitation to its exploitation at an industrial level is its high variability depending on the biomass origin, but several strategies to mitigate these effects have been suggested [31,32].

To this aim, the first part of the study focused on optimising a polymer formulation combining PEO, lignin, and LiTFSI for the subsequent production of nanofibrous electrospun membranes. Building on previous studies in the literature, different polymer solvents, concentrations, and ratios have been assessed [10,28,29,33,34].

The ESP process and parameters are significantly affected by the physical and chemical properties of the spinning solutions, where the selected solvent plays a pivotal role in the successful creation of the polymeric jet and fibres. Compared to other solvents more commonly used in the ESP process such as chloroform, *N*,*N*-dimethylformamide (DMF), formic acid, and dimethyl sulfoxide (DMSO) [33,35,36,37], acetonitrile was selected for this study as a less harsh and greener organic solvent and thanks to its capacity to properly solubilise both PEO and lignin at high concentrations, while guaranteeing high volatility and thus proper evaporation during the process [38]. Besides the selection of the solvent, polymer concentrations and ratio require proper optimisation by defining the threshold values below which the polymeric system is not processable, resulting in the formation of non-fibrous and inhomogeneous membranes characterised by poor stability.

The experiments conducted by varying the solvents, polymer concentration, and the PEO/lithium salt ratio have led to identifying the best conditions to ensure the processability of the spinning solution, which results in a PEO concentration of 10 *w*/*v*% in acetonitrile and a PEO/LiTFSI ratio of 10:1. Indeed, lower polymer concentrations and the use of alternative solvents reported in previous studies led to limited jet stability and poor process reproducibility (Table S1, Figure S1) [28,29,33].

Lignin was subsequently added to the optimised PEO-LiTFSI solution aiming at preserving the physical and chemical properties of the final polymer blend and the stability of the spinning process while improving the stiffness of the electrospun membrane. The final PEO/lignin ratio was optimised to be 9:1 based on the following rheological analysis and maintaining a high content of PEO to preserve the ionic conductivity.

Rheological tests were conducted in order to optimise the material formulation while preliminarily assessing its processability with the ESP process, in comparison with the original PEO-LiTFSI system.

**Figure 2:**
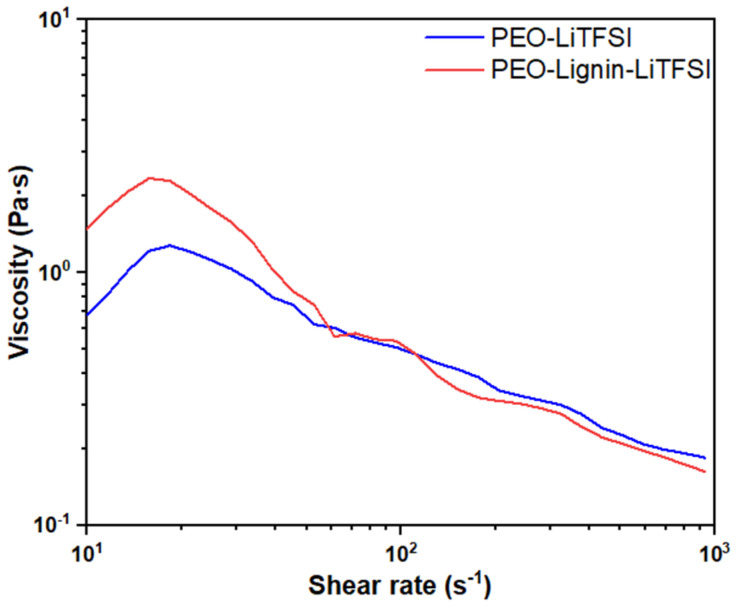
Viscosity curves of PEO-LiTFSI and PEO-Lignin-LiTFSI solutions (PEO/lignin = 9:1).

Figure 2 illustrates the variation in solution viscosity at increasing shear rate values between 10 s^−1^ and 1000 s^−1^. A slight increase in viscosity is observed between 10 s^−1^ and 60 s^−1^, with the maximum value increasing from about 1.28 Pa·s in the PEO-LiTFSI solution to about 2.36 Pa·s following the addition of lignin. The formulation continues to display pseudo-plastic behaviour, with a decrease in viscosity as the shear rate increases, reaching a value of about 0.16 Pa·s at 10^3^ s^−1^. The low values of viscosity registered during the test are consistent with the range of processability that is considered feasible via the ESP of polymeric formulations, i.e., 0.8 Pa·s and 4 Pa·s, hence validating the feasibility of both polymeric solutions [18,39,40]. The obtained results demonstrated that the addition of lignin does not significantly influence the rheology of the final material formulation.

**Figure 3:**
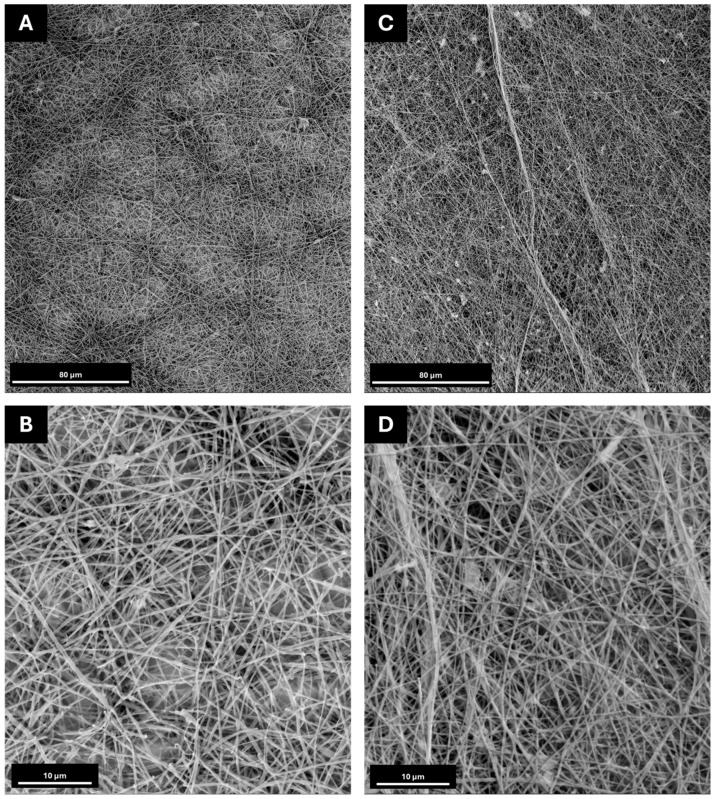
SEM images at 1000× and 5000× magnifications: (**A**,**B**) PEO-LiTFSI at a flow rate of 500 µL/h and a voltage of 18 kV and −2 kV on the collector; (**C**,**D**) PEO-Lignin-LiTFSI at a flow rate of 500 µL/h and a voltage of 15 kV and −2 kV on the collector.

For the electrospinning tests, the jet stabilisation was obtained for each formulation by varying the applied voltage and material flow between 15–19 kV and 0.3–2 mL/h, respectively, using a plate collector and constantly monitoring both the temperature and relative humidity inside the working chamber. The resulting membranes were then collected and analysed using scanning electron microscopy (SEM) to define their morphological features, as shown in Figure 3. Moreover, to determine the potential effect of salt addition on the solution’s processability and the morphological features of the electrospun membrane, solutions of PEO and PEO-Lignin were also processed via ESP. The resulting membranes were then compared to the ones containing salts (Figure S2).

As visible in Figure 3, both formulations result in the production of nanofibrous membranes, despite exhibiting some defects, such as beads, which can be attributed to the incomplete evaporation of the solvent. The average diameter of the fibres remains relatively constant, ranging from 350 ± 130 nm in the PEO-LiTFSI system to 330 ± 120 nm in the PEO-Lignin-LiTFSI system. Consequently, it can be concluded that the incorporation of lignin does not induce alterations in the morphologies of the electrospun membranes. The investigation further revealed that the presence of lithium salt has the most significant impact on the morphology and dimensions of the fibres. The mean diameters of the various types of membranes have been compared in Table S2 of the Supplemental Materials. A comparison of membranes produced exclusively from PEO and the PEO-Lignin mixture (see Figure S2) with those incorporating LiTFSI demonstrates that the addition of the latter results in the formation of less homogeneous membranes, characterised by the presence of flat fibres with a reduced diameter. Despite the slight variation in the morphological features of the membrane, the introduction of the salt did not hinder the overall stability of the ESP process. In contrast, the incorporation of lignin does not result in any alteration to the nanofibre morphology and size, as illustrated in Table S2. In contrast, the incorporation of lignin does not affect the nanofibre morphology or size, as shown in Table S2. This is attributable to the lower lignin concentration relative to PEO. Nonetheless, its presence is important for imparting mechanical strength to the membrane and enabling its handling. Furthermore, as shown in Figure 3, the random distribution creates a densely intertwined structure potentially facilitating the passage of ions and consequently improving the mechanical and electrochemical performance [10,29].

Following the definition of the best ESP parameters (Table 1) to obtain the deposition of homogeneous fibrous membranes, PEO-LiTFSI and PEO-Lignin-LiTFSI were processed up to 4 h to reach a final thickness of 60 ± 2 μm to ensure proper material removal from the aluminium support and further handling. At this stage, the stabilising effect of lignin was already qualitatively evident, since the membrane produced with PEO-Lignin-LiTFSI exhibited better handling and stability compared to the one produced without lignin (PEO-LiTFSI), which was difficult to remove from the substrate and demonstrated poor stability.

### 3.2. Physico-Chemical and Thermal Characterisation of SPEs

**Figure 4:**
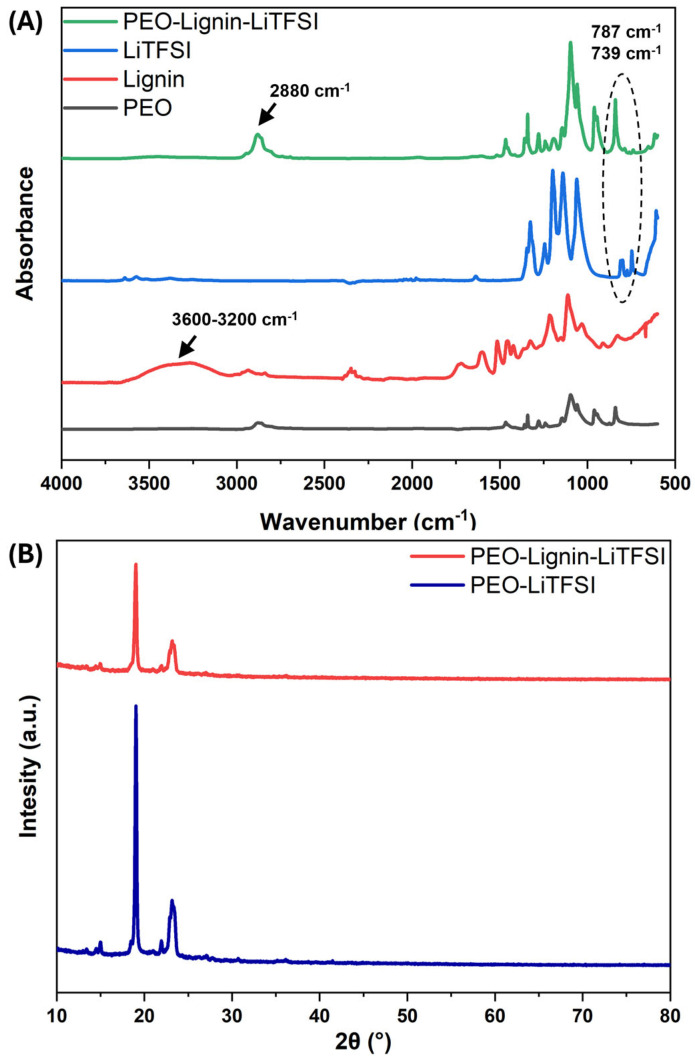
(**A**) ATR-FTIR spectra of pure materials (PEO, lignin, LiTFSI) compared to PEO-Lignin-LiTFSI membrane. (**B**) XRD patterns of PEO-LiTFSI and PEO-Lignin-LiTFSI membranes.

The resulting electrospun SPEs were thoroughly characterised to examine their physico-chemical and thermal properties and the potential variations induced by the presence of lignin. A comparative analysis was conducted on the ATR-FTIR spectra of the complete PEO-Lignin-LiTFSI system and those of each formulation component (i.e., PEO, lignin, and LiTFSI). The results obtained from this analysis are presented in Figure 4A, within the wavenumber range of 4000–600 cm^−1^. Since PEO is the main component of the membrane, its bands are clearly discernible in the spectrum of the mixed PEO-Lignin system, i.e., peaks at 2880 cm^−1^, 1467−1341 cm^−1^, 1278−1240 cm^−1^, 1095 cm^−1^, 961 cm^−1^, and 840 cm^−1^ corresponding to -CH_2_ symmetric and asymmetric stretching, -CH_2_ wagging mode, -CH_2_ twisting mode, C-O-C stretching, and -CH_2_ symmetric and asymmetric rocking, respectively. In particular, the band associated with -CH_2_ symmetric and asymmetric stretching appears to be shifted relative to its position at 2876 cm^−1^ in the spectrum of pure PEO, thus confirming the complexation of the lithium salt with the polymer [10,29]. Meanwhile, the contribution of the lithium salt is given at 787 cm^−1^ and 739 cm^−1^, for the Li^+^TFSI^−^ ion pair and TFSI^−^, respectively. In accordance with other studies, the presence of lignin in the chemical formulation is identified by bands at 2935 cm^−1^ and 2838 cm^−1^ ascribed with the stretching of the -CH_2_ groups while the vibration of the aromatic skeleton is attributed to the bands at 1600 cm^−1^, 1512 cm^−1^, and 1453 cm^−1^ [39,41,42]. The broad band between 3600 cm^−1^ and 3200 cm^−1^, visible in the lignin spectra, is attributed to the H-bonded hydroxyls of the functional groups exposed by the polymer chains. This band in the mixed PEO-Lignin-LiTFSI system, in agreement with previous studies, should be further shifted to a lower wavenumber region due to the formation of a significant number of hydrogen bonds between the two polymers [39,41,42], thus strengthening the overall stability of the membrane. However, the band itself and its shift are barely perceptible in the mixed systems due, as expected, to the limited amount of lignin compared to PEO.

XRD analysis was performed to characterise the crystallinity of the manufactured solid electrolytes, as the presence of a crystalline phase has been demonstrated to influence the conductivity [43]. As shown in Figure 4B, characteristic peaks of PEO are present in the 2θ degree ranges of 19° to 20° and 23° to 24°, illustrating the partially crystalline structure of the fabricated membrane. According to the literature, PEO electrolytes produced using the solvent casting technique exhibit two additional peaks in the 2θ-degree region at 25–30°, indicative of higher crystallinity [10]. Conversely, the absence of these peaks can be attributed to the utilisation of ESP as a production technique. Indeed, the resultant membranes possess a high surface area/volume ratio, thereby facilitating the rapid evaporation of the solvent and thus preventing the formation of unwanted crystalline phases by the polymer chains and salt particles [10,29]. The absence of other significant peaks indicates that the addition of lignin does not lead to the formation of any additional crystalline state. The crystallinity percentage of the membranes was calculated using Equation (1), which revealed that PEO-LiTFSI has a crystallinity percentage of approximately 33.4%, whereas a value of 28.5% was calculated for the PEO-Lignin-LiTFSI system. The incorporation of lignin has been shown to reduce the crystalline phase (as typically observed in polymer blends, in which the addition of a second polymer hinders the ordered arrangement of PEO chains [44]), which is advantageous since lithium ions are known to migrate two to three orders of magnitude faster in amorphous PEO regions with respect to crystalline ones [45]. This is due to polymer chains bound within a crystal lattice preventing the mobility of cations bound to the functional groups along the chain backbone. Consequently, reduced crystallinity facilitates enhanced lithium ion transport [11].

**Figure 5:**
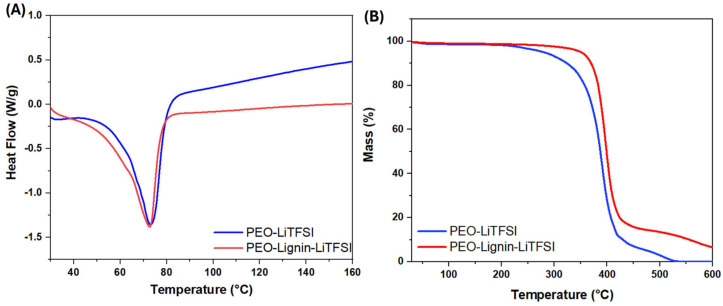
DSC thermograms (**A**) and TGA (**B**) of PEO-LiTFSI and PEO-Lignin-LiTFSI electrospun membranes.

The thermal behaviour of the ESP membranes was evaluated via differential scanning calorimetry (DSC) and thermal gravimetric analysis (TGA) (reported in Figure 5).

The DSC was performed for a range of temperatures between 25 °C and 350 °C, applying a heating rate of 10 °C/min under a nitrogen atmosphere. However, as illustrated in Figure 5A, it is possible to restrict the observation zone to between 30 °C and 160 °C. Both membranes display a main endothermic process at 73 °C, corresponding to the melting point (T_m_), despite some minor processes already starting at a lower temperature (visible as a shoulder at ca. 62 °C in the two DSC profiles). Typically, the T_m_ for PEO-Li_x_ composites is around 60 °C [27]. The higher T_m_ for the PEO-LiTFSI and PEO-Lignin-LiTFSI samples may be attributed to the melting of oriented crystals that are formed during the electrospinning process [46]. The extensional flow aligns polymer chains in the fibre direction, and these extended chains exhibit a higher melting point in comparison to systems produced using conventional techniques [47].

The TGA was conducted at temperatures ranging from 20 °C to 600 °C to study the thermal decomposition behaviour of the developed membranes, which is crucial for developing effective and safe solid electrolytes [18]. As can be seen in Figure 5B, both before and after lignin addition, the membrane degradation process takes place in a single step. The decomposition of the PEO-LiTFSI system begins at approximately 250 °C, reaching a maximum at around 400 °C, resulting in a substantial mass loss. The PEO-Lignin-LiTFSI electrolyte displays analogous behaviour; however, the decomposition process initiates at around 300 °C and continues with a significant percentage mass loss up to 450 °C. In conclusion, the analysis indicates that both electrolytes demonstrate a mass loss of approximately 80%. However, the presence of lignin is evidenced by the shift in the temperature at which the decomposition process begins, from approximately 50 °C, thereby enhancing the overall stability of the electrolyte membrane.

### 3.3. Electrochemical Characterisation

The electrochemical properties of the membranes were investigated to assess their potential application as fully solid polymer electrolytes in Li-based batteries, evaluating their capability of ion conduction, their stability in the typical working potential range, and their compatibility with lithium metal.

**Figure 6:**
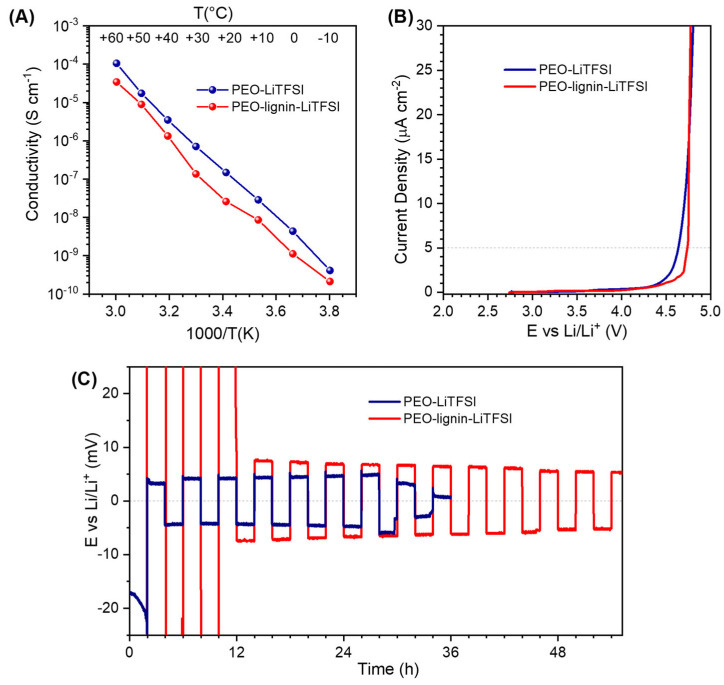
(**A**) Arrhenius plot of ionic conductivity versus inverse temperature determined via EIS in the range from −10 to +60 °C. (**B**) Electrochemical stability window (ESW) of the two solid-polymer electrolyte membranes evaluated via anodic LSV. (**C**) Representative initial cycles extracted from the full test of Li plating and stripping in symmetrical metal-Li cells, with an alternating current of 50 mA cm^−2^ for 2 hours in each verse.

The ionic conductivity of the two solid polymer electrolyte membranes was measured by recording an EIS spectrum every 10 °C, from −10 to +60 °C in two-electrode cell configuration (Figure 6A). For both membranes, it was not possible to measure the conductivity at a temperature higher than 60 °C because both cells underwent a short circuit due to excessive softening of the PEO polymer. Considering all the voids among the fibres, upon the melting of the polymer fraction (in agreement with the DSC analysis shown in Figure 5), the actual amount of material was insufficient to physically separate the opposite electrodes without using a spacer. The conductivity of PEO-LiTFSI varies linearly with the inverse of the temperature, exceeding 10^−4^ S cm^−1^ at the upper 60 °C limit. Such conductivity value is slightly lower than the values typically reported in the literature for much denser PEO-based SPEs obtained by using conventional solvent casting or hot-pressing procedures [48,49,50], but adequately compensated by the area density almost one order of magnitude lower with respect to PEO bulk (1.2 g cm^−3^). Typically, in PEO-based membranes, a change in the slope at ca. 40–50 °C is observed due to the melting of crystalline domains; therefore, the absence of this feature confirms the result of XRD analysis (Figure 4B), which indicates that electrospinning favours the formation of mostly amorphous PEO within the fibres (amorphous portion increases even more when lignin is added in the formulation, likely because of its plasticising effect due to strong hydrogen bonding with the ether oxygen of PEO and/or to the conformational affinity between the chain structures) [51]. In addition, in the presence of lignin, the conductivity of the membrane is only slightly lower, which is nonetheless not expected to compromise its behaviour in electrochemical cells and can be compensated for by the increase in mechanical strength. It is worth remarking that the conductivity values do not consider the voids among the fibres, which make the membranes extremely lightweight (ca. 4 mg cm^−2^), and potentially suitable for batteries with a high gravimetric energy density.

The anodic stability of the samples was measured via LSV in two-electrode cells with Li metal as counter and quasi-reference electrode (QRE) and carbon-coated Al as working electrode (Figure 6B). The current limit for determining the anodic stability of the membranes was set to 5 μA cm^−2^, to fulfil the commercial requirements [52]. The addition of lignin positively affects the electrochemical stability of the membrane, which is slightly increased up to 4.75 V vs. Li/Li^+^, compared to the membrane of PEO-LiTFSI that starts the oxidative degradation at a lower potential, around 4.6 V vs. Li/Li^+^.

Both the electrospun polymer electrolyte membranes were tested in symmetric Li|Li cell, to evaluate their electrochemical behaviour upon Li plating and stripping at 55 °C, and the representative initial cycles of the test are shown in Figure 6C. At the very beginning, the PEO-Lignin-LiTFSI membrane displayed a higher overpotential (ca. 0.5 V) but then stabilised with an overpotential comparable with PEO-LiTFSI (±8 mV). Moreover, the PEO-Lignin-LiTFSI membrane resisted for a much longer time, while the cell with PEO-LiTFSI underwent a short circuit quite soon, proving that lignin confers to the membrane a higher resistance against dendrite growth and the consequent battery breakdown.

## 4. Conclusions

In this study, electrospinning technique was successfully adopted to prepare thin and flexible nanofibrous membranes to be tested as solid polymer electrolytes in all-solid-state Li metal batteries. The ESP technique was selected due to its versatility, its ability to finely control the structure and morphology of the produced membranes, and its easy scalability in industrial practice.

Among the polymeric materials that are currently proposed to produce SPEs, PEO was selected due to its extensive application in this field because of its low cost, easy processability, and the possibility to effectively dissolve lithium salts while promoting ionic conductivity. To enhance the mechanical strength and integrity of PEO-based membranes upon electrochemical cycling at higher temperatures, lignin was incorporated as a reinforcing element. In addition, since lignin is the primary byproduct of the paper industry, this approach also provides a way to repurpose a waste polymer into a high-value-added application, promoting the production of SPEs within a circular economy model.

The successful electrospinning of nanofibrous PEO-lignin-LiTFSI membrane was here achieved through a step-by-step optimisation of the polymeric formulation. The rheological analysis confirmed the appropriate processability of the formulation, while the morphological assessment of the electrospun membranes allowed the identification of the ESP process parameters needed to achieve homogeneous nanofibres throughout the membranes. Structurally, the addition of lignin in the formulation contributes to reducing PEO crystallinity by obstructing the arrangement of the chains, which positively encourages the application of PEO-Lignin-LiTFSI membrane in LIBs because Li^+^ movement is favoured in amorphous PEO. Moreover, the PEO-Lignin-LiTFSI system exhibited significant stability and ease of handling.

Finally, as a proof of concept, both the PEO-LiTFSI and PEO-Lignin-LiTFSI membranes were tested in symmetric Li|Li electrochemical cell at 55 °C (lower temperature than the melting point), demonstrating that both membranes are compatible with reversible Li plating and stripping and that the addition of lignin is effective in contrasting the Li dendrite growth and hence preventing the consequent battery breakdown for a longer time.

As a future perspective, to boost the application of the developed SPE in industrial practice, further optimisation will be needed to fulfil the strict requirements of battery manufacturing, allowing for a more stable cycling in electrochemical cells and possibly the evaluation of a post-processing treatment aimed at the chemical crosslinking of polymer chains.

Overall, these promising results display the potential benefits of the ESP technique to produce easily tunable SPEs, blending different polymers with suitable properties and revalorising waste materials. Moreover, the achieved outcomes could pave the way for using this fabrication technology in real-world applications, particularly for specific targets such as ultrathin, lightweight all-solid-state micro-batteries.

## Figures and Tables

**Table 1:** ESP process parameters to produce PEO-LiTFSI and PEO-Lignin-LiTFSI membranes.

	Distance Tip to Collector (cm)	Flow Rate (µL/h)	Voltage (kV)	Voltage on Collector (kV)
**PEO-LiTFSI**	18	500	18	−2
**PEO-Lignin-LiTFSI**	18	500	15	−2

## Data Availability

All the data are contained within the main text of the article and the Supplementary Materials.

## Supplementary Materials

The following supporting information can be downloaded at: https://www.mdpi.com/article/10.3390/polym17070982/s1, Table S1: Composition of tested formulations; Figure S1: SEM images at 1000× and 5000× magnifications: (A,B) 5% w/v PEO in Acetonitrile; (C,D) PEO-Lignin-LiTFSI in DMSO: Acetone; Figure S2: SEM images at 1000× and 5000× magnifications: (A,B) PEO; (C,D) PEO-Lignin; Table S2: Average size of diameters determined using ImageJ software; Figure S3: EIS spectra of PEO-LiTFSI and PEO-lignin-LiTFSI solid polymer electrolyte membranes in symmetric cells with stainless-steel blocking electrodes at 15 °C. The equivalent circuit used to fit the spectra is shown, where Rel is the resistance value used to calculate the conductivity reported in Figure 6.
