# Supplementary material for: The Impact of Recovered Lignin on Solid-State PEO-Based Electrolyte Produced via Electrospinning: Manufacturing and Characterisation
Source: Polymers (Basel). 2025 Apr 4;17(7):982. doi: 10.3390/polym17070982 (PMC11991043; doi:10.3390/polym17070982)
Supplement: Supplementary file 1 [file polymers-17-00982-s001.zip › polymers-3553693-supplementary.pdf]

SUPPORTING INFORMATION

The impact of recovered lignin on solid-state PEO-based electrolyte produced by electrospinning: manufacturing and characterization

Laura Coviello [1], Giorgia Montalbano [1], Alessandro Piovano [1,2], Nagore Izaguirre [3], Chiara Vitale Brovarone [1], Claudio Gerbaldi [1,2], Sonia Fiorilli [1]

[1] Department of Applied Science and Technology (DISAT), Politecnico di Torino, Corso Duca Degli Abruzzi 24, 10129 Torino, Italy.
[2] National Reference Centre for Electrochemical Energy Storage (GISEL)–INSTM, Via Giusti 9, 50121 Firenze, Italy.
[3] Chemical and Environmental Engineering Department, Engineering Faculty of Gipuzkoa, University of the Basque Country UPV/EHU, Plaza Europa 1, 20018 Donostia, Spain.

In accordance with extant literature, the optimisation of the formulation was pursued through the experimental testing of varying concentrations of PEO and solvents [1–3]. The composition of tested formulation is reported in Table S1 and morphology is showed in Figure S1.

Table S1. Composition of tested formulations

	PEO concentration	Ratio Lignin:PEO	Ratio LiTFSI:PEO	Solvent
**A-B**	**5%(w/v)**	**-**	**-**	**Acetonitrile**
**C-D**	**20% (w/v)**	**1:9**	**1:10**	**DMSO:Acetone (70:30)**

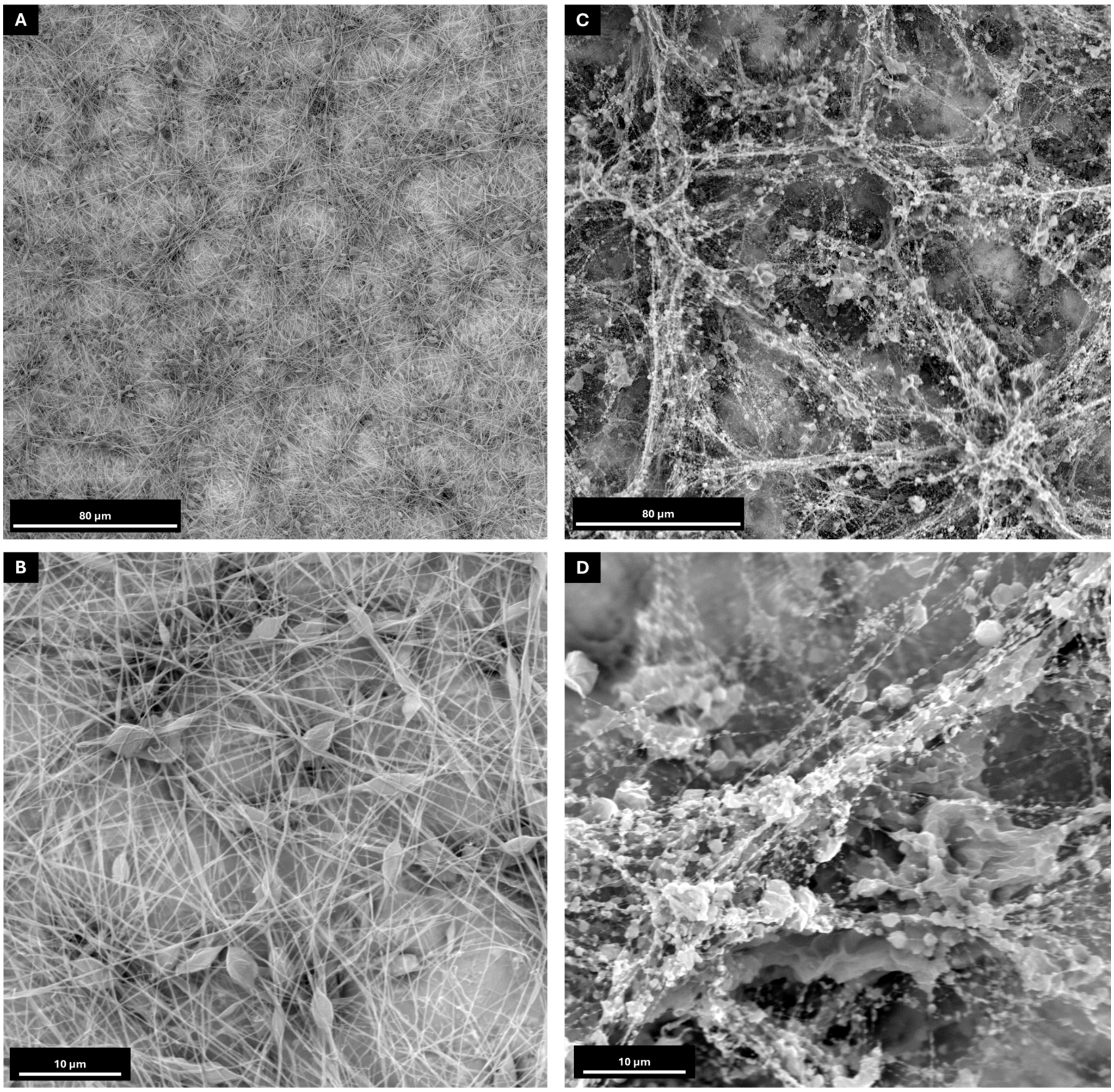

Figure S1. SEM images at 1000X and 5000X magnifications: A-B) 5% w/v PEO in Acetonitrile; C-D) PEO-Lignin-LiTFSI in DMSO : Acetone.

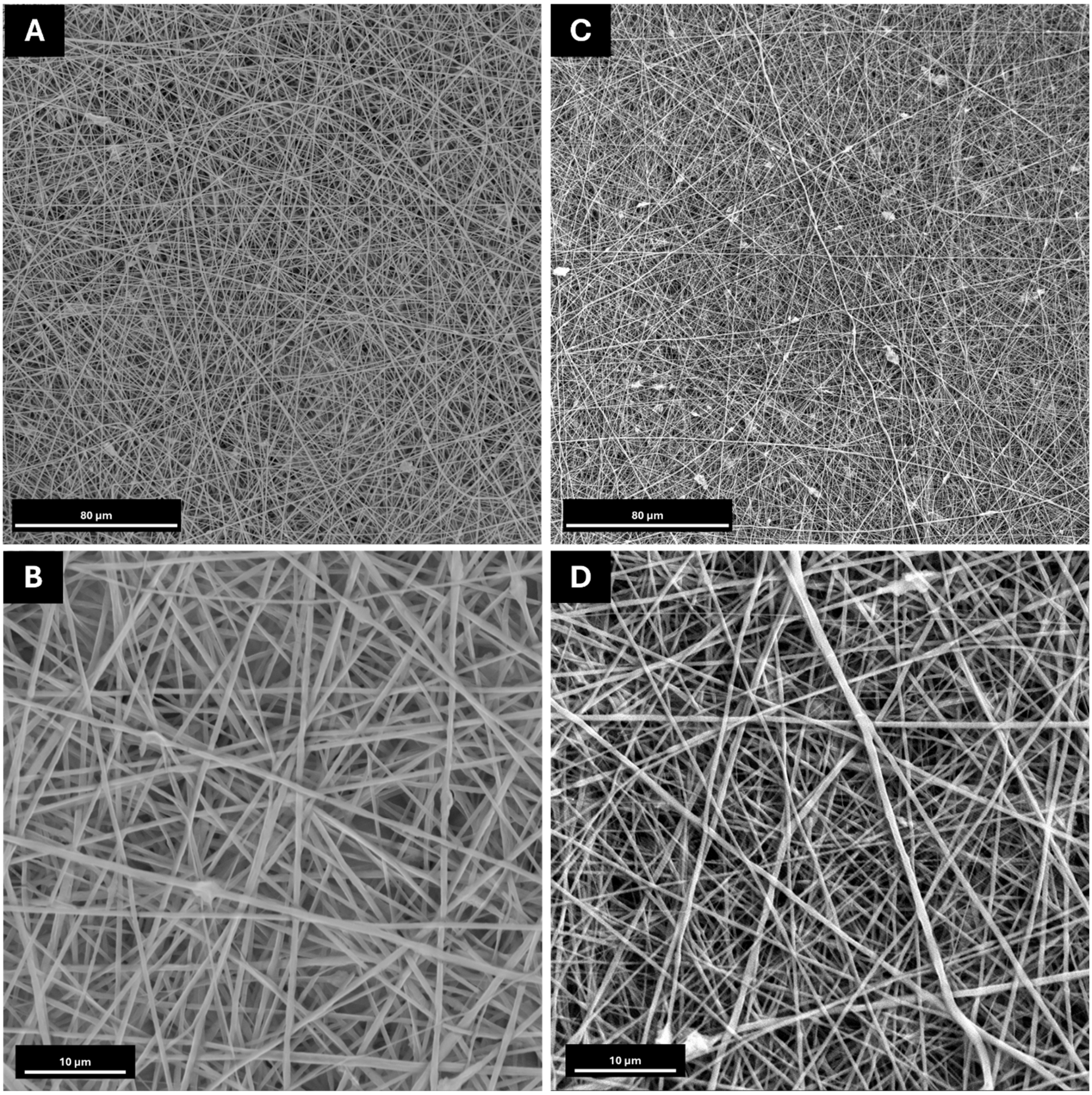

Figure S2. SEM images at 1000X and 5000X magnifications: A-B) PEO; C-D) PEO-Lignin.

Table S2. Average size of diameters determined using ImageJ software

	**Average diameter (nm)**
**PEO**	490 ± 200
**PEO-Lignin**	440 ± 230
**PEO-LiTFSI**	350 ± 130
**PEO-Lignin-LiTFSI**	330 ± 120

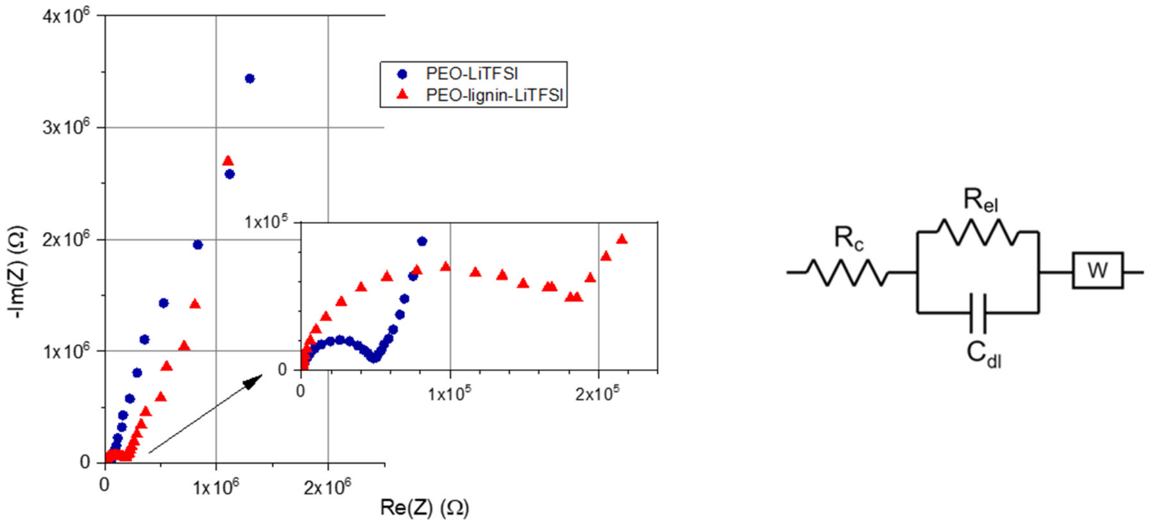

Figure S3. EIS spectra of PEO-LiTFSI and PEO-lignin-LiTFSI solid polymer electrolyte membranes in symmetric cells with stainless-steel blocking electrodes at 15 °C. The equivalent circuit used to fit the spectra is shown, where R_{el} is the resistance value used to calculate the conductivity reported in Figure 6.

1. Aslanzadeh, S.; Ahvazi, B.; Boluk, Y.; Ayranci, C. Morphologies of Electrospun Fibers of Lignin in Poly(Ethylene Oxide)/N,N-dimethylformamide. J of Applied Polymer Sci 2016, 133, app.44172, doi:10.1002/app.44172.
2. Banitaba, S.N.; Semnani, D.; Karimi, M.; Heydari-Soureshjani, E.; Rezaei, B.; Ensafi, A.A. A Comparative Analysis on the Morphology and Electrochemical Performances of Solution-Casted and Electrospun PEO-Based Electrolytes: The Effect of Fiber Diameter and Surface Density. Electrochimica Acta 2021, 368, 137339, doi:10.1016/j.electacta.2020.137339.
3. Walke, P.; Freitag, K.M.; Kirchhain, H.; Kaiser, M.; Van Wüllen, L.; Nilges, T. Electrospun Li(TFSI)@Polyethylene Oxide Membranes as Solid Electrolytes. Zeitschrift anorg allge chemie 2018, 644, 1863–1874, doi:10.1002/zaac.201800370.
